# Oleuropein mediated autophagy begets antimalarial drug resistance

**DOI:** 10.3389/fmicb.2024.1453998

**Published:** 2024-08-20

**Authors:** Prakriti Sharma, Neil Roy Chaudhary, Sonia Devi, Sushmita Negi, Nikunj Tandel, Rajeev K. Tyagi

**Affiliations:** ^1^Division of Cell Biology and Immunology, Biomedical Parasitology and Translational-Immunology Lab, CSIR Institute of Microbial Technology (IMTECH), Chandigarh, India; ^2^Academy of Scientific and Innovative Research (AcSIR), Ghaziabad, India; ^3^Institute of Science, Nirma University, Ahmedabad, Gujarat, India

**Keywords:** malaria, autophagy, *P. falciparum*, drug resistance, oleuropein, humanized mouse model

## Abstract

Drug resistance in *Plasmodium falciparum* presents a formidable challenge to the humanity. And, unavailability of an effective vaccine worsens the situation further. Autophagy is one of the mechanisms employed by parasite to evade drug pressure to survive. Autophagy induced by the *P. falciparum* in response to the oleuropein pressure may answer many questions related to the parasite survival as well as evolving drug tolerance. The survival/autophagy axis could be an important avenue to explore in order to address certain questions related to the evolution of drug resistance. In addition, humanized mouse model of *P. falciparum* infection could serve as an important preclinical tool to investigate the oleuropein-induced autophagy, potentially helping to dissect the mechanisms underlying the development of antimalarial drug resistance.

## 1 Introduction

*P. falciparum*, the human malaria parasite, gaining tolerance/resistance to antimalarial drugs and poses a significant challenge for treating malaria infection. The failure of monotherapy begets advent of artemisinin-based combination therapy (ACTs) to treat malaria infection (Mutabingwa, 2005; Nguyen et al., 2023). The parasite however has been gaining resistance to even ACTs and present a significant threat for humanity (Tyagi et al., 2018a). The situation becomes critical in the wake of unavailability of an effective malaria vaccine. Furthermore, there have been reports suggesting that drug induced autophagy drives the evolution and spread of antimalarial drug resistance (Ray et al., 2022; Sharma et al., 2024). The development of drug tolerant parasites threatens the effectiveness of malaria treatment strategies and present a significant challenge. Further, a key obstacle in addressing the drug resistance is the complex mechanisms underlying the antimalarial drug resistance especially in *P. falciparum* (Wicht et al., 2020). The development of resistance phenotypes within parasite populations, characterized by genetic mutations in molecular drug targets and enhanced efflux pump activities, leads to the diminished antimalarial drug accumulation (Shibeshi et al., 2020). This in turn results in a progressive tolerance to the sub-therapeutic drug dosages. Such evolutionary dynamics complicate therapeutic strategies and undermine the clinical effectiveness of current antimalarial drug therapies (Wicht et al., 2020). Furthermore, the spread of drug tolerant mutant parasites across regions exacerbates the global burden of malaria. Hence, this necessitates continuous surveillance and innovation in drug development to prevent parasites from gaining resistance to drugs (Ippolito et al., 2021). Autophagy is one of the key mechanisms employed by the parasite to evade drug pressure (Ray et al., 2022; Pandit et al., 2023; Sharma et al., 2024). The ring stage parasite invasion is crucial for the development of parasite (Joy et al., 2018). Antimalarial drug treatment inhibits the parasite reinvasion. Hence, autophagy employed by the parasite helps parasite to tolerate therapeutic effects of drugs and slowly gain resistance to the antimalarials (Rubinsztein et al., 2007; Kannan et al., 2023). The upregulation of autophagy pathways in response to the antimalarial drug exposure may enhance parasite survival by facilitating nutrient acquisition, promoting cellular remodeling, and evading host immune responses (Leleu et al., 2022; Schroeder et al., 2022). Additionally, autophagy-mediated mechanisms, such as the degradation of drug compounds or alteration of drug targets, can contribute to the development of drug resistance in malaria parasites. Therefore, the interplay between autophagy and drug resistance could be crucial for devising novel therapeutic strategies. Thus, targeting these pathways may be essential to surmount the challenges posed by the drug-tolerant *P. falciparum* strains, with the ultimate goal of enhancing treatment modalities for human malaria parasites.

## 2 Induction of autophagy by the anti-inflammatory oleuropein

Oleuropein (OLP), a potent natural compound found in olive leaves, has garnered attention for its diverse health benefits (Nediani et al., 2019). Recent research has unveiled its remarkable ability to induce autophagy during *P. falciparum* infection to survive drug pressure (Sharma et al., 2024). The role of OLP in inducing autophagy not only expands our understanding of its anti-inflammatory properties but also opens therapeutic avenues for understanding evolving antimalarial drug resistance. Present paper delves into the intricate interplay between the oleuropein induced autophagy and thereby conferred parasite survival. This could be useful in developing novel therapeutic interventions for human malaria infection.

This phyto-compound OLP exhibits anti-inflammatory, antioxidant, and immunomodulatory properties, along with the ability to induce autophagy (Rigacci et al., 2015; Lins et al., 2018; Cirmi et al., 2022; Sharma et al., 2024). To investigate the effect of OLP, an *in vitro* model was developed utilizing human THP-1 macrophages stimulated with an antigen (LPS). This model was used to determine the anti-inflammatory and PI3K-Akt1 signaling pathways regulated by OLP (Sharma et al., 2024). Also, OLP was reported to induce cell death in cancer cells (Asgharzade et al., 2020). Therefore, we investigated programmed cell death in response to the antigen stimulation in the human macrophages (THP-1 cells). We confirmed the antimalarial activity, induction of autophagy and attested the signaling *in vitro, in silico* (molecular docking), and in the challenge model of *P. berghei* infection. Recently we discovered that OLP may regulate antigen-induced cell death through the CD40 pathway and the PI3K-Akt1 signaling cascade (Sharma et al., 2024). OLP treatment appears to influence the balance between cell death and autophagy, potentially preventing the clearance of the antigen-stimulated cells due to the stress following the OLP treatment (Liu et al., 2019; Sharma et al., 2024). Cells treated with OLP showed a suppression of phosphorylated NF-kβ (Rel), and hence checking the cell death apoptotic pathways (Yamamoto and Gaynor, 2001). Overall, we confirmed the activation of autophagy in response to OLP treatment by the altered gene and protein expression related to immune response, apoptosis, and autophagy in the *in vitro* macrophage model of inflammation (MMI). We had confirmed the antimalarial activity of OLP in the different laboratory strains (3D7, Dd2, and D10-Atg18) in the routine asexual blood stage infection of *P. falciparum*, and also monitored the parasite growth. Besides, we analyzed the autophagy-related protein (ARP) and development-related protein (DRP) expression aiming at confirming the activation of autophagy like escape mechanism employed by macrophages to develop resistance to the apoptosis (Sharma et al., 2024). Further, our molecular docking analyses suggest the interactions between OLP, artesunate (ART), and proteins associated with autophagy and cellular development indicating the activation of autophagy. Our findings with the challenge model of *P. berghei* infection showed that OLP treatment exhibited the inhibited parasite growth with the altered gene expression of *mdr1* (drug resistance) and *Atg8* (autophagy) levels, particularly at the lower OLP concentrations (Sharma et al., 2024). Moreover, the histopathology assessed the changes in the cell infiltration, whereas ELISA performed on OLP treated MMI that exhibited the modulation of LPS induced inflammation characterized by the estimation of IL-6 (pro-inflammatory) and IL-10 (anti-inflammatory/immunoregulatory). In a nutshell, OLP is shown to drive the host to activate autophagy during malaria infection by influencing the cell death/autophagy axis. This helped parasite survive for extended periods under physiological stress or drug pressure. The study conducted by Sharma *et al*. demonstrated that OLP treatment did not show significant parasite clearance in the routine *in vitro* cultures of *P. falciparum*. On the contrary, OLP enhancing the antimalarial activity of ART during the combination therapy indicating the additive effect important for parasite clearance (Sharma et al., 2024). Based on our findings, we believe activation of autophagy by the host may help parasite develop tolerance toward antimalarial drugs. The gene transcription and protein expression analysis of ARPs and DRPs confirmed the activation of autophagy for evading the OLP pressure. The possibility of conducting combination studies with existing antimalarial drugs to evaluate potential synergies in to hinder the evolution of drug resistance. Therefore, OLP could be partner drug in combination with ART to increase the antimalarial potential of ART. In essence, OLP induces autophagy in malaria parasites when submitted to the drug pressure and enable parasite develop tolerance toward the therapeutic effect of antimalarial drugs. Present study investigated the antimalarial potential of OLP alone and in combination with ART to treat malaria infection as well as showed the possibility of combating the evolution and spread of drug resistance in malaria (Sharma et al., 2024).

### 2.1 Autophagy in malaria parasite

Autophagy plays a crucial role in maintaining cellular homeostasis and could contribute to the development of antimalarial drug resistance. Through this lysosome-mediated degradation pathway, malaria parasites can modulate their cellular environment to counteract the pharmacodynamic effects of antimalarial compounds, thereby promoting their survival and propagation under drug-imposed selective pressures. There are reports that suggest the increased levels of autophagy in the artesunate-resistant *P. falciparum* parasites (Ray et al., 2022). Additionally, chloroquine-resistant parasites exhibit a more pronounced induction of autophagy compared to that with the chloroquine-sensitive parasites (Kamil et al., 2022). The drug-resistant parasites utilize autophagy to enhance the survival by facilitating the degradation of damaged organelles and proteins, reducing oxidative stress, and improving nutrient uptake (Coppens, 2011; Ray et al., 2022). Understanding the molecular underpinnings of autophagy in *P. falciparum* is thus essential for the development of innovative therapeutics targeting this intracellular pathway.

### 2.2 Autophagy and survival of malaria parasite

The survival of the malaria parasite hinges on its ability to manipulate host cell processes, including autophagy, to ensure its replication and propagation. Based on our findings we believe OLP disturbs the parasite's ability to subvert host defenses and exploit host resources for its benefit. This disruption not only impairs the growth and development of parasite but also makes its susceptibly to the immune defense system of the host (Leleu et al., 2022). The interplay between autophagy and the survival of the malaria parasite illuminates a critical vulnerability that can be targeted for the development of novel antimalarial therapies. Further, OLP inclined the balance in favor of host immunity by modulating the autophagy. It could be important for combating malaria and reducing the global burden of human malaria parasite infection.

Autophagy induction in malaria occurs notably in the sporozoites during metamorphosis (Coppens, 2011). Treatment with 3-methyladenine (3-MA) impacts Vps34 (vacuolar sorting proteins), a key player in the parasite, leading to a significant delay in sporozoite differentiation (Coppens, 2011). Within *Plasmodium* genomes, recognizable orthologs of ATGs (autophagy-related genes), such as ATG1, ATG17, ATG18 in the ATG9 cycling system, ATG12 in the ATG12 conjugation system, and ATG3, ATG4, ATG7, and ATG8 in the ATG8 conjugation system, are present. The similarity in ATG orthologs between human malaria parasite *P. falciparum* and rodent parasite *P. berghei* ranges from 50 to 87%. Beyond organelle degradation via autophagy, *Plasmodium'*s Atg8 facilitates vesicular trafficking during the intrahepatic development's anabolic phase (Coppens, 2011).

Antimalarial drugs exploit autophagy mechanism to combat the parasite infections, yet their efficacy diminishes with the development of drug tolerance in *P. falciparum*. *Pf* ATG plays a crucial role in autophagosome formation, retrieval, and vesicle breakdown. *Pf* ATG8, a marker for phagophores and autophagosomes, localizes to the apicoplast membrane during late schizonts and merozoites. Apicoplasts, non-photosynthetic plastids, have evolutionary origins in endosymbiosis. *Pf* ATG8, a phosphatidylethanolamine-conjugated protein, envelops vesicles throughout sexual and asexual erythrocytic stages, augmenting the conventional/classic secretory channel for exporting parasite proteins to host RBCs via vesicular transport (Banerjee et al., 2012; Agrawal et al., 2020).

*In vitro* findings advocating autophagy induction during antigen stimulation in MMI were validated in a rodent model of *P. berghei* infection. Remarkably, early parasite clearance withstood OLP pressure, evading elimination in later stages, indicative of host-driven autophagy activation. Transcription profiling of signaling, inflammatory, and autophagy markers confirmed parasite autophagy utilization. Aligning with previous studies (Joy et al., 2018; Walczak et al., 2018; Sharma et al., 2024), targeting autophagy pathways emerges as a promising strategy to address the issue of antimalarial drug resistance. Inhibiting autophagy in artemisinin-resistant parasites could restore treatment efficacy (Ray et al., 2022). And, combining autophagy inhibitors with antimalarial drugs effectively cleared parasites in *P. falciparum* cultures and rodent models. Nonetheless, the role of autophagy in drug resistance necessitates careful consideration of dosages and timings of drug and the administration of autophagy inhibitor (Chakraborty, 2016).

Very recently, we explored the potential of OLP in regulating inflammation and affecting autophagy to counter the anti-plasmodial defenses. The investigation focuses on the relevance of OLP in malaria therapy, considering the challenges posed by drug resistance and the absence of effective vaccines. Our findings shed light on the dynamic interplay among OLP, autophagy, and parasite survival, offering valuable insights for advancing malaria research and developing therapeutic interventions for malaria treatment (Sharma et al., 2024).

### 2.3 Challenge model of *P. falciparum* infection (humanized mouse model) to investigate the OLP induced autophagy

Rodent malaria parasites such as *P. yoelii* and *P. berghei* are used as surrogates for studying *P. falciparum*. Therefore, immunodeficient (NSG) mice reconstituted with huRBCs is an ideal challenge model of *P. falciparum* infection (Arnold et al., 2011; Tyagi et al., 2018b). Leveraging rodent parasites, which share the syntenic arrangement with *P. falciparum*, offers a valuable tool to investigate human malaria parasites.

**Figure 1:**
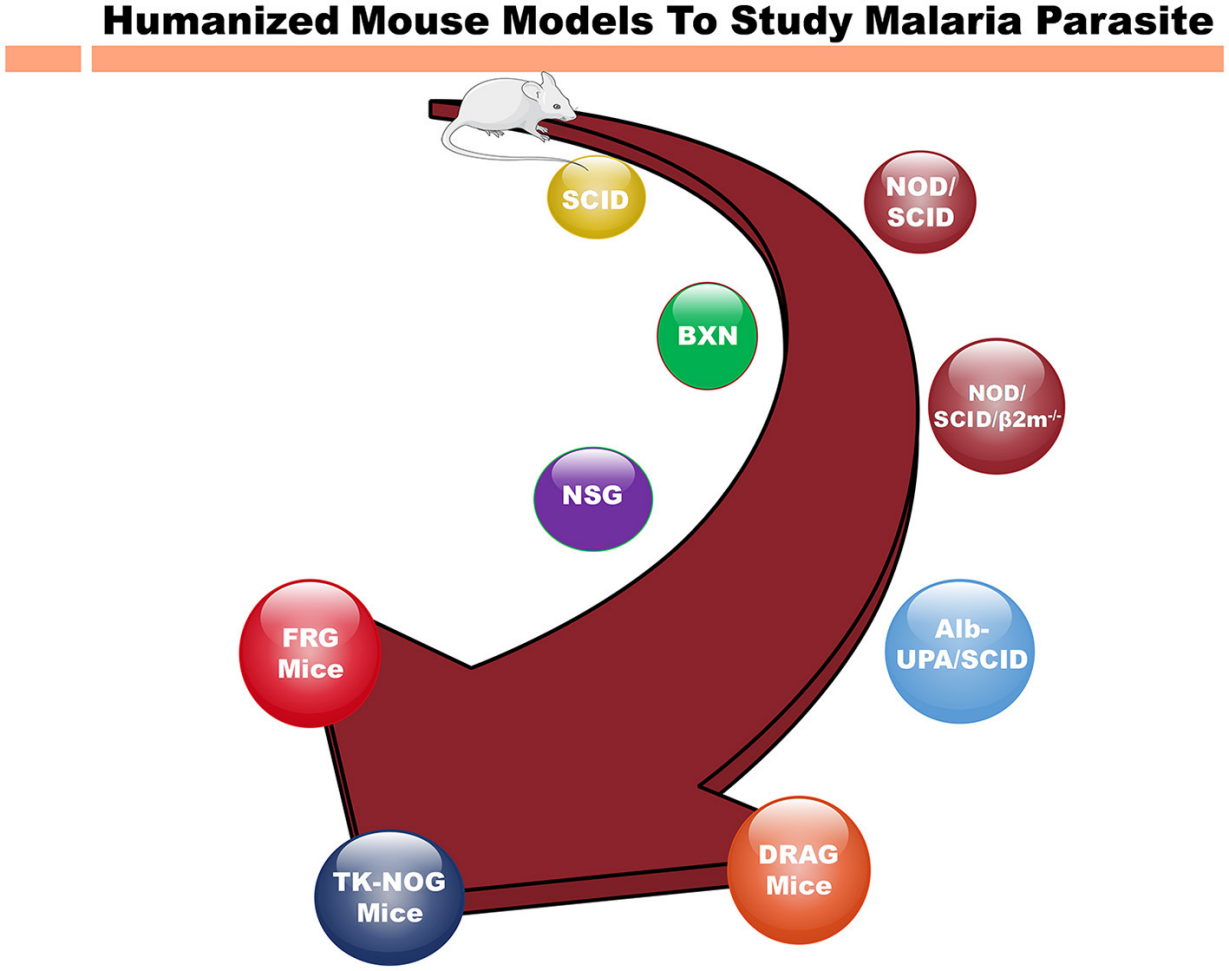
Different immunodeficient mouse strains for humanization to study human malaria parasite. Evolution of immunodeficient mouse models from the original SCID (severe combined immunodeficient) mice to highly immunocompromised strains for humanization to study infectious, inflammatory diseases and cancer. As the time progressed, immunodeficient mice such as NSG (NOD scid gamma) mice that support the human cell grafting. The newer immunodeficient mouse strains such as FRG and TK-NOG mice, represent the latest technologies based on genetic engineering increased the immunodeficiency of immune cells to study the human diseases. The SCID mice, lacking functional T and B cells due to a DNA repair enzyme gene mutation were found crucial into study the immune system. Over the periods of time, more immunocompromised mouse strains such as NSG mice with no NK cells in addition to other immunodeficiency were engineered. These developments are invaluable for investigating human pathologies, advancing drug development, and refining immunotherapies. Mouse strains, such as FRG and TK-NOG, with other genetic modifications allowed to develop human liver chimeric mice to study liver stage (LS) infection of *P. falciparum*. The NOD/SCID/β_2_m^−^ variant, lacking MHC class I due to an additional β_2_m gene knockout, hinders the functionality of immune system, and hence promoting xenotransplantation. NSG mice, with scid and IL2rg mutations, completely lack T, B, and NK cells, and are widely employed in xenografting and human immune system reconstitution studies. The NSG mice reconstituted with hematopoietic stem cells (CD34^+^) are show to recapitulate the whole immune system and these mice are famously referred to as humanized immune system mice. Also, Alb-UPA/SCID mice, bearing a UPA (urokinase-type plasminogen activator) transgene, induce liver damage for the human hepatocyte engraftment to investigate the liver diseases. DRAG mice, with SCID mutations and human immune system components, facilitate research into human immune responses. Finally, FRG mice, with their triple knockout (Fah^−/−^, Rag2^−/−^, Il2rg^−/−^) has been a crucial mouse model for developing human liver chimeric mice to investigate the infectious disease (Hepatitis B, LS infection of *P. falciparum*) as well as to study the pharmacokinetics and metabolism of human-specific liver-metabolized drugs.

Nevertheless, the interspecies differences underscore necessity for the humanized mouse models to comprehensively explore the biology, pharmacology and immunology of *P. falciparum* (Arnold et al., 2011). Thus, the reconstitution of immunodeficient mouse strains with human cells (Table 1) present a more accurate representation to mimic the human malaria infections (Arnold et al., 2011; Tyagi et al., 2018a,b; Tyagi, 2021; Figure 1).

**Table 1:** Immunodeficient mouse strains used for the humanization to study asexual blood and live stage infection of *P. falciparum* (adapted and modified from Zhang et al., 2009; Tyagi et al., 2018b).

**Mouse strain**	**Mechanism**	**Characteristics**	**References**
BXN (Beige/Xid/Nude)	Beige, Xid, and Nude mutation	Deficiency in functional NK cells, T cells, and T-cell independent B cells	Novak et al., 1980
SCID	DNA-repair and VDJ recombination defect due to deficiency of DNA-PK	T and B cell deficiency	Mccune et al., 1988
SCID/beige	Combined effect of SCID and beige mutations	T and B cell deficiency	Mosier et al., 1993
NOD/SCID	VDJ recombination defect added to various NOD anomalies	T and B cell deficiency	Prochazka et al., 1992
NOD/SCIDA/γc^null^	Combined effect of SCID and γc^null^ on NOD background	Severe defect of T and B cell function	Ito et al., 2002
Rag^null^	VDJ recombination defect	T and B cell deficiency	Mombaerts et al., 1992; Shinkai et al., 1992; Chen et al., 1994
NOD/LtSz-Rag1^null^Pfp^null^	VDJ recombination defect and perforin mutation	T and B cell deficiency	Shultz et al., 2003
Rag2^nu^γc^null^ mice	Combined effect of Rag2^null^ and γc^null^	T and B cell deficiency	Colucci et al., 1999
FRG (FAH^−/−^Rag2^−/−^IL2Rγ^null^)	Immune-deficient Fah knockout	Rag-2 and gamma chain of the interleukin receptor deficient	Azuma et al., 2007; Bissig et al., 2007
TK- NOG	HSV Tk gene construct	UL23 or HSVtk gene expressed	Hasegawa et al., 2011; Soulard et al., 2015
DRAG (HLA-DR4.RagKO.IL2RγcKO.NOD)	Combination of HLA class II antigen binding domain molecules with the I-E^d^ MHC-II	Engraftment of human hematopoietic stem cells, development of human B and T cells, enable to reconstitute all the human immunoglobulins and their subclasses, and able to generate the specific antibodies against the vaccination	Wijayalath et al., 2014

SCID, severe combined immunodeficiency; NOD, non-obese diabetes; TK/NOG, thymidine kinase/NOD/Shi-scid/IL-2Rγnull mic.

The intraperitoneal (IP) injection of AB^+^ human serum was shown to extend the half-life of huRBCs in SCID mice following intravenous (IV) injection (Tsuji et al., 1995). Furthermore, SCID mice have been preconditioned with irradiation prior to the challenge with *P. falciparum*, promoting the stable integration of huRBCs (Tsuji et al., 1995). The residual innate immune responses were suppressed by the immunomodulatory agents, such as dichloromethylenebisphosphonate-(Cl_2_MBP)-encapsulated liposomes (Badell et al., 1995). The employment of AB^+^ huRBCs has also been reported to achieve better blood-chimerism in SCID/NIH III mice. Additionally, the administration of human cytokines has been utilized to enhance huRBCs engraftment, whereas infusion of CD34^+^ hematopoietic stem cells (HSCs) into gamma-irradiated NSG mice, supplemented with a plasmid encoding erythropoietin and human IL-3 was used to enhance the engraftment of human leukocytes and huRBCs (Chen et al., 2009). These comprehensive approaches showed a higher engraftment index for huRBCs, support the blood stage infection of *P. falciparum* and to study the antimalarial potential of novel drugs. We developed the optimal blood-stage mouse model (huRBCs-NSG-IV; Arnold et al., 2011) by controlling the residual innate immune responses. Clodronate-loaded liposomes deplete 70–80% of monocyte/macrophage. Also, we validated its value of this mouse model by experimentally inducing resistance to artesunate (Tyagi et al., 2018a).

The human cells (huRBCs and huHep) reconstituted NSG/TK-NOG mice allowed to study the liver stage infection and transition from liver to the blood stage infection of *P. falciparum* (Tyagi et al., 2018b).

Autophagy has been studied in the rodent model of *P. berghei* infection (Leleu et al., 2022; Sharma et al., 2024) in order to understand the role of autophagy to confer survival on parasite to sustain the host responses (Prado et al., 2015; Thieleke-Matos et al., 2016). These studies have provided valuable insights into the complex interplay between autophagy, the malaria parasite, and the host immune system. However, limitations include species-specific differences, genetic variability, complex host-parasite interactions, ethical concerns, and challenges in to accurately mimic the human disease (Tyagi, 2021; Simwela and Waters, 2022). We believe the complementary methods like humanized mice and *in vitro* models can enhance our understanding of autophagy in malaria, and hence aid in developing more effective antimalarial strategies.

**Figure 2:**
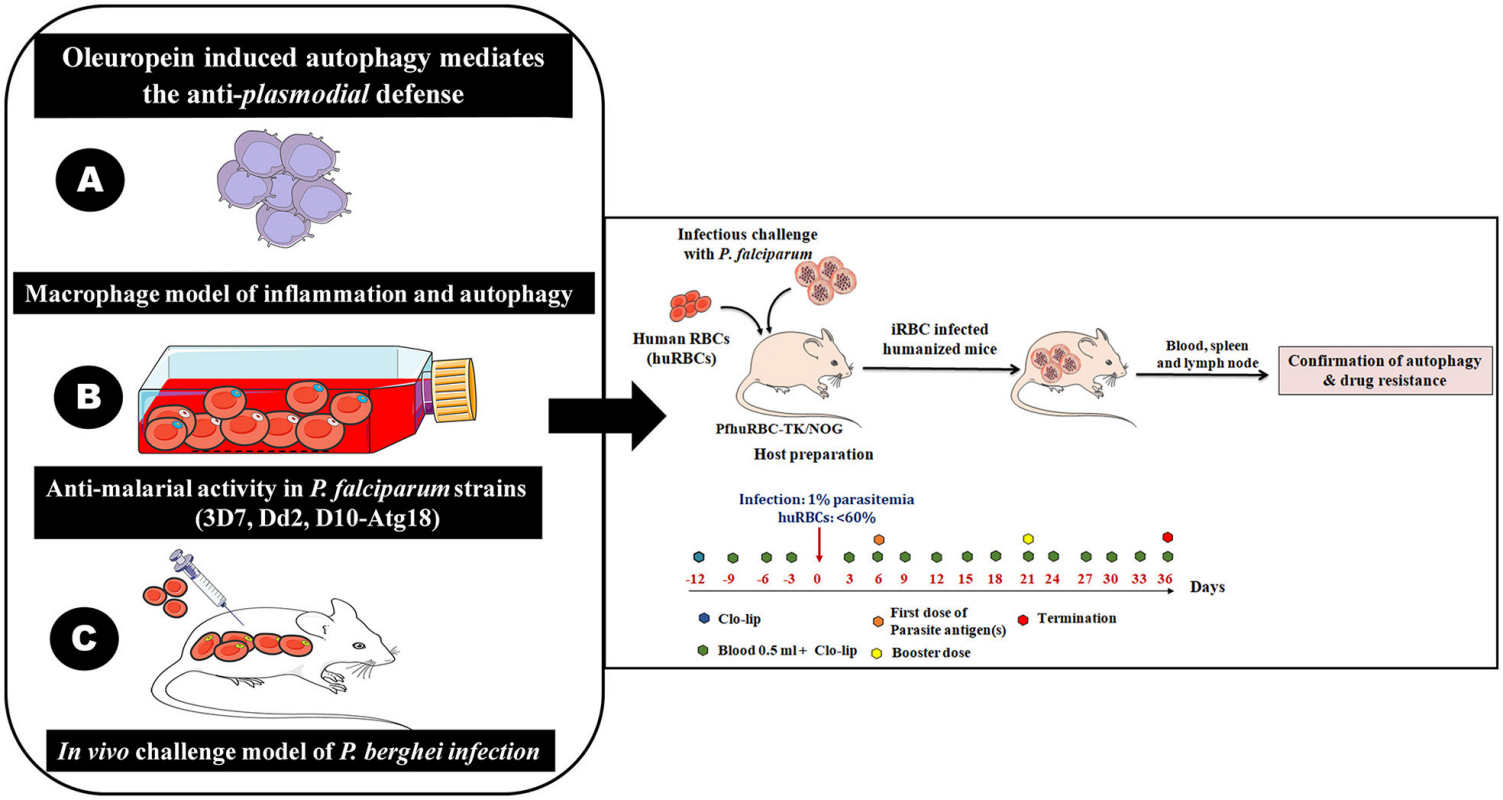
The immunomodulatory protocol for developing human red blood cells reconstituted NOD. Prkdcscid *Il*2*rg*^−/−^ (NSG/TK-NOG) mice for *P. falciparum* infection to study autophagy. It is a multi-step experimental design to study OLP-induced autophagy and its role in mediating anti-plasmodial defenses. The role of OLP on malaria parasites and activation of autophagy (by host) to circumvent anti-plasmodial defense. **(A)** Establishment of a lipopolysaccharide (LPS)-mimicked and PMA differentiated human THP-1 macrophages (Macrophage model of inflammation; MMI) to study the cellular mechanism through which OLP affects the immune response. **(B)** Assessment of the antimalarial activity of OLP in three laboratory *P. falciparum* strains (3D7 and D10 parasites expressing GFP-tagged Atg18, chloroquine-resistant Dd2). The antimalarial activity and induction of autophagy like survival mechanism studied at gene transcription and protein expression levels. The induced autophagy was tightly controlled by the Akt1-mediated signaling pathways. **(C)** Development of challenge model of *P. berghei* infection to attest our *in vitro* findings. Righ panel; sequential steps for the *in vivo* experiments involving infectious challenge model of *P. falciparum*. The timeline showed days of interventions: administration of Clo-lip (a formulation of liposomes containing a drug named clodronate). Administration of 0.5 ml along with Clo-lip formulation. The timeline also specifies the preparation of huRBCs reconstituted NSG/TK-NOG mice by administering four injections prior to give infectious challenge at 1% asynchronous parasite.

We confirmed the antimalarial activity of OLP in combination with artemisinin as well as activation of autophagy like survival mechanism under OLP pressure in the rodent model of *P. berghei* infection (Figures 2A–C). Therefore, the challenge model of *P. falciparum* infection is essential to attest our findings. Hence, we propose the development of humanized moue model harboring *P. falciparum* to investigate the role of OLP in autophagy and its potential as an anti-malarial agent. We may reconstitute huRBCs in NSG mice and give infectious challenge with different laboratory strains of *P. falciparum* strains and study the therapeutic aspects of OLP (Figure 2-Right panel). The humanized mouse model provides a platform for assessing the *in vivo* relevance and possible therapeutic effects of OLP against malaria. This model shall enable us studying the how OLP influences host to activate autophagy and how latter contributes to the development and spread of antimalarial drug resistance. Also, host response to OLP pressure could be studied that shall eventually address on how parasite escapes drug pressure. In essence, humanized mouse model(s) of *P. falciparum* infection could be a valuable preclinical *in vivo* tool to study drug therapeutics and dynamics as well as host's response to drug pressure. In the end, this integrative approach enhances our understanding about the modulation of autophagy that may open areas for developing novel anti-malarial drugs.

## 3 Implications of autophagy in antimalarial drug resistance

Autophagy activation in malaria parasites, induced by physiological stress or drug pressure may explain antimalarial drug resistance in humans. This could aid in developing new drugs and drug targets to address resistance. Our research indicates that targeting autophagy might prevent resistance, offering new antimalarial strategies. However, these findings need validation in the challenge model of *P. falciparum* infection. Such models are crucial for studying newer drugs to confirm their antimalarial potential as well as understanding the role of autophagy in sustenance and bypassing the host immunity. Overcoming technical challenges, such as model variability and the lack of high-throughput screening for autophagy modulators, is necessary for further developments. Future malaria research will likely prioritize autophagy modulation, alongside other key areas like vaccines and vector control. The integration of autophagy-targeting drugs with current antimalarial therapies is anticipated, potentially revolutionized by the humanized mouse models and CRISPR-Cas9 gene-editing technology.

OLP study have significant clinical implications, particularly in the context of the urgent need for new developing newer therapeutic strategies to combat malaria infection and address antimalarial drug resistance. Managing drug resistance is quite challenging due to the emergence of drug resistant parasite, which can rapidly spread, leading to treatment failures and increased mortality rates. Surveillance is crucial in monitoring the emergence and spread of drug-resistant parasites, providing critical data on the effectiveness of current treatments and guiding the development of newer drugs and treatments. Ongoing surveillance systems are essential for identifying drug resistance mutations and population dynamics of *P. falciparum*. For instance, the study by Moss et al. (2023) highlights the importance of understanding population dynamics and drug resistance mutations in *P. falciparum* on the Bijagos Archipelago, Guinea-Bissau (Moss et al., 2023). Similarly, Ward et al. (2023) identified a multidrug resistance-1 (MDR-1) mutation associated with increased *in vitro* susceptibility to mefloquine in *P. cynomolgi*, underscoring the need for continued surveillance and genetic analysis. New drug development is pivotal in the fight against drug resistance. The study by Sharma et al. suggests that OLP could be a promising partner drug in artemisinin based combination therapy to clear malaria infection. The findings also indicate that OLP has antimalarial activity when the parasite is exposed to the drug for longer durations, suggesting its potential use as an antimalarial drug. This may lead to the development of new antimalarial drugs or drug combinations that inhibit autophagy employed by parasite, thereby enhancing the efficacy of existing treatments. The drug-induced autophagy could be instrumental in to develop effective malaria vaccines and treatment strategies.

## 4 Conclusion and future perspectives

Understanding OLP, its therapeutic effect and its ability to induce autophagy in *P. falciparum* could be crucial for developing therapeutic approaches for malaria infection. Hence, the interplay between autophagy, inflammation, and parasite survival gives direction for the development of novel antimalarial therapies. Our recent findings wherein we explored OLP in the macrophage model of inflammation, the routine culture of *P. falciparum* infection, lefts an additive effect to increase the anti-malarial activity of artesunate when used in combination. The *in vitro* findings were validated in the rodent model of *P. berghei* infection (Sharma et al., 2024).

## Data Availability

The original contributions presented in the study are included in the article/supplementary material, further inquiries can be directed to the corresponding authors.
